# *FADS1-FADS2* and *ELOVL2* gene polymorphisms in susceptibility to autism spectrum disorders in Chinese children

**DOI:** 10.1186/s12888-018-1868-7

**Published:** 2018-09-04

**Authors:** Caihong Sun, Mingyang Zou, Xuelai Wang, Wei Xia, Yongjuan Ma, Shuang Liang, Yanqiu Hao, Lijie Wu, Songbin Fu

**Affiliations:** 1 Department of Children’s and Adolescent Health, Public Health College, Harbin Medical University, Harbin, 150081 China; 2 Department of Pediatric, The Second Affiliated Hospital, Harbin Medical University, Harbin, 150081 China; 3 Laboratory of Medical Genetics, Harbin Medical University, Harbin, 150081 China

**Keywords:** Autism spectrum disorders, *FADS1–2; ELOVL2*, Long-chain polyunsaturated fatty acids, Single nucleotide polymorphisms

## Abstract

**Backgroud:**

Autism spectrum disorders (ASD) are a complex group of neurodevelopmental disorders with a genetic basis. The role of long-chain polyunsaturated fatty acids (LC-PUFAs) and the occurrence of autism has been the focus of many recent studies. The present study investigates whether genetic variants of the *fatty acid desaturase* (*FADS*) 1/2 and *elongation of very long-chain fatty acids protein* (*ELOVL*) 2 genes, which are involved in LC-PUFA metabolism, are associated with ASD risk.

**Methods:**

A cohort of 243 ASD patients and 243 unrelated healthy controls were enrolled in this case control study. Sixteen tag single nucleotide polymorphisms from the *FADS1–2* and *ELOVL2* genes were genotyped using the Sequenom Mass Array.

**Results:**

There were significant differences in allelic distribution of *FADS2 rs526126* (OR = 0.55, 95% CI = 0.42–0.72, *p*_*FDR*_ < 0.05) between autistic children and controls. *FADS2 rs526126* and *ELOVL2 rs10498676* were associated with decreased ASD risk in recessive model (OR = 0.07, 95% CI = 0.02–0.22, *p*_*FDR*_ < 0.01; OR = 0.56, 95% CI = 0.35–0.89, *p*_*FDR*_ = 0.042), while *ELOVL2 rs17606561*, *rs3756963*, and *rs9468304* were associated with increased ASD risk in overdominant model (OR = 1.63, 95% CI = 1.12–2.36, *p*_*FDR*_ = 0.036; OR = 1.64, 95% CI = 1.14–2.37, *p*_*FDR*_ = 0.039; OR = 1.75, 95% CI = 1.22–2.50, *p*_*FDR*_ = 0.017). The A/A genotype of *rs10498676* was correlated with a decline in the Autism Diagnostic Interview-Revised communication (verbal and nonverbal) domain.

**Conclusions:**

These findings provide evidence of an association between *FADS2* and *ELOVL2* polymorphisms and ASD susceptibility in Chinese children.

**Electronic supplementary material:**

The online version of this article (10.1186/s12888-018-1868-7) contains supplementary material, which is available to authorized users.

## Background

Autism spectrum disorders (ASD) are a heterogeneous group of neurodevelopmental disorders characterized by abnormal social interaction, impaired language and communication, and repetitive stereotypic behaviors or a narrow range of interests [1]. ASD likely result from complex interactions between multiple genes and environmental factors, with some studies suggesting that genetics play a role in the majority of autism cases [2]. Genomic analyses have revealed that single nucleotide polymorphisms (SNPs) in specific genes are associated with predisposition to ASD and may explain the phenotypic variability observed in patients [3].

**Fig. 1:**
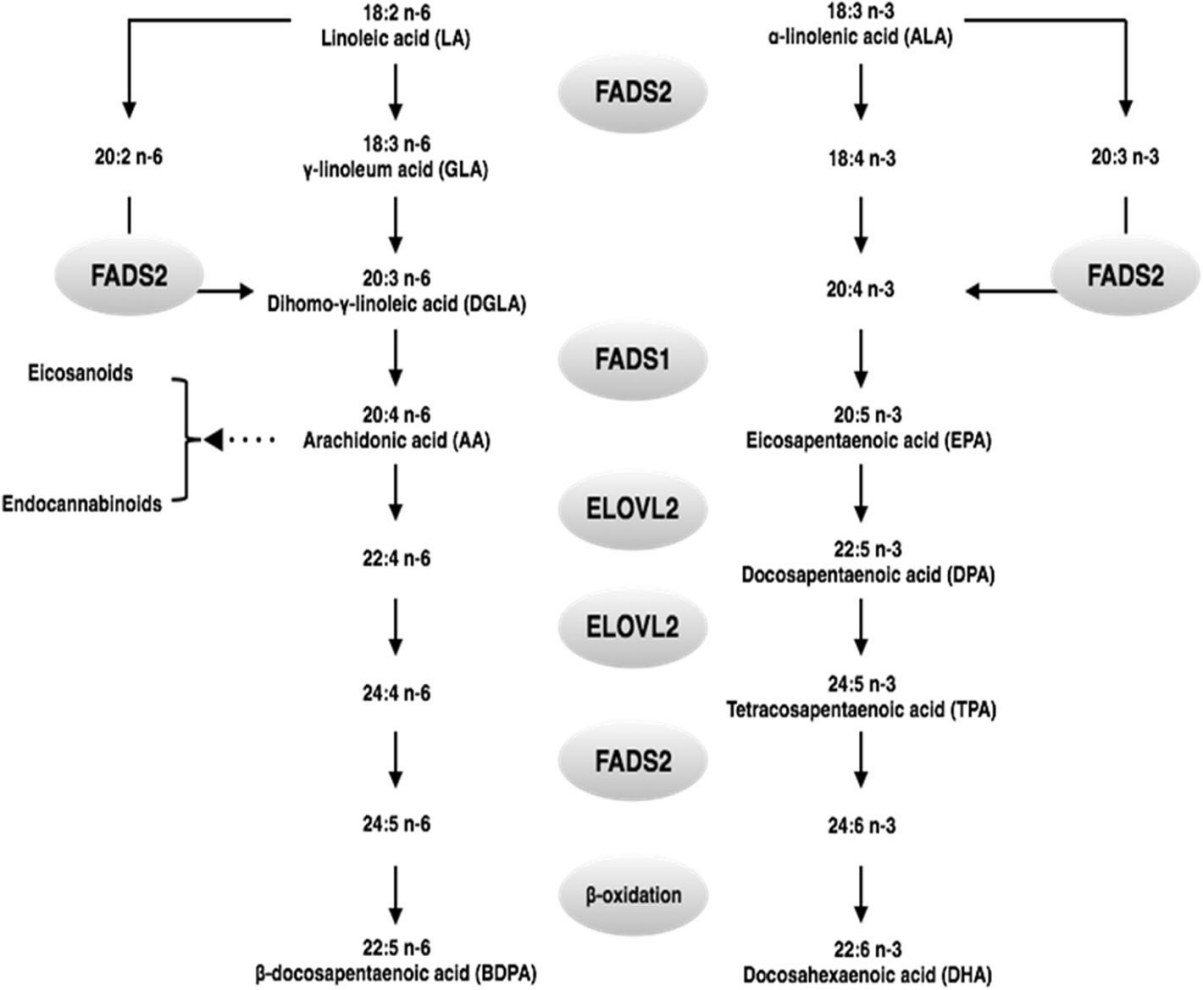
Metabolic pathways for endogenous n-6 and n-3 polyunsaturated fatty acids

Polyunsaturated fatty acid (PUFAs) are classified as omega-3 (n-3) or omega-6 (n-6) depending on the location of the last double bond relative to the terminal methyl group in the carbon chain [4]. The essential PUFA precursors linoleic acid (LA, 18:2n-6) and alpha-linolenic acid (ALA, 18:3n-3) are metabolized by elongation and desaturation into long-chain (> 20 C atoms) (LC-) PUFAs, such as arachidonic acid (AA, 20:4n-6), eicosapentaenoic acid (EPA, 20:5n-3), and docosahexaenoic acid (DHA, 22:6n-3). The two types of PUFA share and compete for the same enzymes in their biosynthesis (Fig. 1). Rate-limiting LC-PUFA biosynthetic enzymes in humans include delta-5 and -6 desaturase, and elongases 2 encoded by *fatty acid desaturase* (*FADS*)*1*, *FADS2*, and *elongation of very long-chain fatty acids protein* (*ELOVL*)*2*, respectively. Independent of dietary effects, polymorphisms in these genes have been shown to affect LC-PUFA status in different ethnic groups [5–9]. LC-PUFAs are indispensible components of neuronal membranes and modulate the integrity, fluidity, and function of transmembrane proteins. They also act as second messengers in intracellular signaling pathways or regulate gene transcription and expression, neurogenesis, neuroprotection, and neurotransmission, and serve as precursors for the synthesis of eicosanoids and docosanoids, which are potent regulators of immune and inflammatory processes in the brain [4, 10]. In particular, DHA and AA, the principal LC-PUFAs in the brain, that mediate many physiological effects. Numerous epidemiological and experimental studies have reported a link between LC-PUFA content in blood and certain tissues and mental health as well as cognitive and motor development [7, 11–13].  As such, there is great interest in investigating the potential connection between LC-PUFA levels and the occurrence of autism.

Although there is evidence to suggest that autistic patients have abnormal LC-PUFA metabolism that manifests as a reduction in their levels [14–17], few studies have explored the relationship between genes involved in LC-PUFA metabolism and risk of ASD. *FADS1/2* and *ELOVL2* gene polymorphisms may be the primary determinants not only of LC-PUFA status, but also of the biological effects exerted by LC-PUFAs [9]. We previously reported that serum LC-PUFA levels and delta-6 and -5 desaturase and elongase 2 were downregulated in a rat model of ASD relative to control rats under the same dietary conditions [18]. These findings indicate that mutations in the *FADS1/2* and *ELOVL2* genes resulting in abnormal expression of delta-6 and -5 desaturase and elongase 2, possibly due to perturbed LC-PUFA status during critical stages of neural development that increases susceptibility to ASD. To clarify the etiology of ASD, the present study investigated the relationship between *FADS1/2* and *ELOVL2* gene polymorphisms and ASD risk as well as clinical features of ASD in a Chinese Han population.

## Methods

### Subjects

A total of 243 consecutive patients with ASD were recruited from the Child Development and Behavior Research Center of Harbin Medical University, Harbin, China, from May 2013 to March 2016. Inclusion criteria were a diagnosis of ASD, which was made by a specialist clinician according to Diagnostic and Statistical Manual of Mental Disorders, Fifth Edition (DSM-V) criteria. One of the following methods was used as an aid to diagnose: Childhood Autism Rating Scales, Autism Behavior Checklist, Autism Diagnostic Observation Schedule (ADOS), Autism Diagnostic Interview-Revised (ADI-R). Exclusion criteria were children with significant sensory and motor impairment, known genetic disorders, seizures at the time of enrollment, and any other neurological conditions suspected to be associated with autism. Unrelated healthy children (*n* = 243) without history of developmental delay or other neurological disorders were randomly selected from normal kindergartens in Harbin, China as the controls. All study subjects were of Chinese Han ethnicity.

### Clinical assessment

Autism-specific behaviors and symptoms were evaluated according to ADOS and ADI-R, which were administered during interviews with patients or their guardians and scored by a clinical psychologist or trainee who met standard requirements for research reliability. Due to applicability, cooperativity and compliance from patients and their guardians, some cases did not complete above two examinations. Only 184 of 243 ASD cases completed the ADOS and/or ADI-R. The ADOS was available for 174 assessments (71.6% of sample), the ADI-R for 176 assessments (72.4%). Nine participants were excluded due to missing data. Sample characteristics are provided in Additional file 1: Table S1.

ADOS is a semi-structured standardized observational assessment that is organized into four modules according to developmental and expressive language level. The calibrated severity score (CSS), which is a standardized score of the relative severity of autism-specific behaviors, is less influenced by participant demographics than by raw scores [19]. The ADOS CSS algorithm encompasses social affect (SA) and restricted repetitive behavior (RRB) domains, which are consistent with DSM-V [20]; these allow comparisons of assessments across modules and times. Procedures for deriving the ADOS-CSS, SA-CSS and RRB-CSS are detailed in the original study [19, 21].

ADI-R is a semi-structured parent interview that assesses autism symptoms across three domains: social, communication, and repetitive behavior. Although the algorithm scores were originally designed to provide categorical diagnostic information, taking language level and age at the time of interview into account, ADI-R domain and total scores can be used as estimates of the severity of core ASD symptoms [22]. Higher adjusted ADI-R scores indicate greater impairment. Before similar strategies used in ADOS can be applied to ADI-R, ADI-R scores must be statistically adjusted for language level indicated by the ADOS module, intelligence quotients (IQ), sex and age. The IQ scores were derived from the Wechsler Preschool and Primary Scales of Intelligence, Wechsler Intelligence Scale for Children or the Peabody Picture Vocabulary Test.

### SNP selection and genotyping

We used genotyping data from the Chinese Han in Beijing (CHB) panel of the HapMap Project Phase II to select tag SNPs in *FADS1/2* and *ELOVL2* genes using the Tagger program incorporated with Haploview v.4.2 software (Broad Institute of Massachusetts Institute of Technology and Harvard, Cambridge, MA, USA) according to the following criteria: pairwise tagging (r^2^ ≥ 0.8) and minor allele frequency ≥ 0.05, leading to the identification of 16 tag SNPs (one in *FADS1* and seven in *FADS2* that captured more than 34 initially identified SNPs; and eight in *ELOVL2 *that captured more than 45 initially identified SNPs). Detailed information for tag SNPs are showed in Additional file 1: Table S2.

Blood samples were obtained from each participant in an EDTA-containing tube. Genomic DNA was extracted using the Qiagen QIAamp DNA Mini kit (Qiagen, Hilden, Germany) according to the manufacturer’s instructions. DNA quality was confirmed on a 1% agarose gel, and DNA concentrations were determined by measuring the absorbance at 260 nm using a NanoDrop 2000 spectrophotometer (Thermo Fisher Scientific, Waltham, MA, USA). SNPs were genotyped using the MassArray platform (Sequenom, San Diego, CA, USA) with the primers showed in Additional file 1: Table S3. For genotyping, investigators were blinded to case/control status, and 30 random samples were tested in duplicate for all 16 tag SNPs for quality control, with a reproducibility of 100%.

### Statistical analyses

Haploview v.4.2 software was used to evaluate deviations from Hardy–Weinberg equilibrium (HWE) among controls, as well as for analysis of linkage disequilibrium (LD) and haplotype construction. SNPstats software (http://bioinfo.iconcologia.net/SNPstats) was used to calculate the odds ratios (ORs) and 95% confidence intervals (CIs) in different genetic models. SNP associated analyses were estimated under the following genetic models: codominant, dominant, recessive, overdominant and log-additive model. When the values of Akaike’ information criterion (AIC) and Bayesian information criterion (BIC) are the lowest, the genetic model is the optimal model for the SNP. Benjamini-Hochberg approach based on false discovery rate (FDR) criterion was applied to avoid the type I error in multiple comparisons. Potential relationships between genetic and phenotypic characteristics were evaluated by one-way analysis of variance with adjustment for language level, IQ, sex and age at the time of assessment, and the *Bonferroni* correction was applied in multiple testing. Data were analyzed using SPSS v.17.0 software (SPSS Inc., Chicago, IL, USA). All reported *p* values were two-tailed, and statistical significance was defined at the α = 0.05 level.

## Results

### Clinical characteristics

The 243 cases included 204 boys and 39 girls with a mean age of 5.19 ± 1.96 years. The 243 controls included 192 boys and 51 girls with a mean age of 4.96 ± 0.97 years. There were no differences in terms of age and gender between cases and controls (*p* = 0.101 and *p* = 0.161, respectively).

### Association between the SNPs and ASD risk

Call rates for the 16 tag SNPs exceeded 95%. These differences in genotyping efficiency were attributable to different sample sizes for each SNP measured. The *rs2845573* was excluded from statistical analyses because the corresponding genotypes in the control group deviated from HWE (*p* < 0.05). The genotype distributions of the remaining SNPs were in agreement with HWE in the control group (*p* > 0.05; Table 1).

**Table 1:** Characteristics of *FADS1–2* and *ELOVL2* tag SNPs

SNP ID	Gene	Genomic position (bp)	Genic position	Reference allele ^a^	Call rate %	MAF ^b^	HWE ^b^ (*p*)
*rs174546*	*FADS1*	61,802,358	3’UTR	C	98.6	0.322	0.958
*rs2845573*	*FADS2*	61,834,436	intron	A	98.8	0.071	< 0.001*
*rs174585*	*FADS2*	61,844,222	intron	G	99.8	0.091	0.786
*rs174593*	*FADS2*	61,851,359	intron	T	100.0	0.095	1.000
*rs174602*	*FADS2*	61,856,942	intron	T	98.3	0.241	0.455
*rs498793*	*FADS2*	61,857,233	intron	T	99.0	0.075	0.266
*rs526126*	*FADS2*	61,857,413	intron	C	96.7	0.428	0.175
*rs174616*	*FADS2*	61,861,650	intron	G	99.8	0.152	0.994
*rs17606561*	*ELOVL2*	10,982,126	3’UTR	G	99.8	0.215	0.580
*rs2236212*	*ELOVL2*	10,994,782	intron	C	98.1	0.332	1.000
*rs3798712*	*ELOVL2*	11,007,869	intron	A	98.8	0.233	0.912
*rs953413*	*ELOVL2*	11,012,626	intron	A	97.9	0.095	0.186
*rs3756963*	*ELOVL2*	11,021,921	intron	T	99.7	0.229	0.492
*rs10498676*	*ELOVL2*	11,026,766	intron	G	98.1	0.450	0.100
*rs6936315*	*ELOVL2*	11,035,739	intron	T	98.8	0.288	1.000
*rs9468304*	*ELOVL2*	11,041,932	intron	A	99.8	0.320	0.408

*MAF* minor allele frequency, *HWE* Hardy-Weinberg equilibrium, * *p* < 0.05

^a^ determined by most frequent allele among controls

^b^ among controls (*n* = 243)

Table 2 summarized the allele frequencies of the SNPs in ASD patients and controls. Allele frequencies for *FADS2 rs526126* and *ELOVL2 rs9468304* differed significantly between cases and controls (*p* < 0.05). The *rs526126* G allele (OR = 0.55, 95% CI = 0.42–0.72, *p* < 0.001) was associated with a lower risk of ASD, whereas the *rs9468304* G allele (OR = 1.37, 95% CI = 1.05–1.79, *p* = 0.035) was associated with a higher risk of ASD. The allele distributions of other tag SNPs were non-significant. However, the positive result of *rs9468304* presented no statistical significance after FDR-based correction.

**Table 2:** Distribution of allelic frequencies of SNPs in cases and controls (n, %)

SNPs	Allele (major:minor)	Control (243)		Case (243)		OR(95%CI)	*p*	*p*_*FDR*_
		major	minor	major	minor			
*rs174546*	C:T	324(67.8)	154(32.2)	328(68.3)	152(31.7)	0.98(0.75–1.29)	0.855	0.916
*rs174585*	G:A	440(90.9)	44(9.1)	452(93.0)	34(7.0)	0.75(0.47–1.20)	0.230	0.493
*rs498793*	C:T	444(92.5)	36(7.5)	444(92.1)	38(7.9)	1.07(0.66–1.72)	0.823	1.029
*rs174602*	T:C	360(75.9)	114(24.1)	375(77.5)	109(22.5)	0.94(0.70–1.28)	0.575	0.958
*rs174593*	T:C	440(90.5)	46(9.5)	451(92.8)	35(7.2)	0.73(0.46–1.16)	0.202	0.505
*rs526126*	C:G	275(56.6)	211(43.4)	315(69.4)	139(30.6)	0.55(0.42–0.72)	**< 0.001**	< 0.05*
*rs174616*	G:A	412(84.8)	74(15.2)	414(85.5)	70(14.5)	0.93(0.65–1.33)	0.738	1.107
*rs17606561*	G:A	380(78.5)	104(21.5)	360(74.1)	126(25.9)	1.31(0.97–1.77)	0.104	0.390
*rs2236212*	C:G	318(66.8)	158(33.2)	317(66.3)	161(33.7)	1.01(0.77–1.32)	0.873	0.873
*rs3798712*	A:G	365(76.7)	111(23.3)	374(77.3)	110(22.7)	0.94(0.69–1.27)	0.828	0.955
*rs953413*	A:G	436(90.5)	46(9.5)	415(88.3)	55(11.7)	1.28(0.84–1.93)	0.280	0.525
*rs3756963*	T:C	373(77.1)	111(22.9)	354(72.8)	132(27.2)	1.28(0.96–1.72)	0.129	0.387
*rs10498676*	G:A	263(55.0)	215(45.0)	289(60.7)	187(39.3)	0.79(0.61–1.02)	0.075	0.375
*rs6936315*	T:C	339(71.2)	137(28.8)	349(72.1)	135(27.9)	0.92(0.70–1.23)	0.760	1.036
*rs9468304*	A:G	329(68.0)	155(32.0)	299(61.5)	187(38.5)	1.37(1.05–1.79)	**0.035**	0.263

*Major* major allele, *minor* minor allele, *OR* Odds ratio, *CI* confidence interval, *FDR* false discovery rate, *p*_FDR_ FDR corrected *p* value, ^*^ *p* < 0.05

We analyzed the association of the SNPs with ASD risk in five genetic models (Additional file 1: Table S4). As shown in Table 3, there were significant differences in genotype frequencies of *rs526126*, *rs17606561*, *rs3756963*, *rs10498676*, and *rs9468304* between the patient and control groups (*p* < 0.05). After the *p* values were adjusted by FDR-based correction, *FADS2 rs526126* (OR = 0.07, 95% CI = 0.02–0.22, *p*_*FDR*_ < 0.01), and *ELOVL2 rs10498676* (OR = 0.56, 95% CI = 0.35–0.89, *p*_*FDR*_ = 0.042) were associated with a reduced risk of ASD in the recessive model. Whereas *ELOVL2 rs17606561* (OR = 1.63; 95% CI = 1.12–2.36, *p*_*FDR*_ = 0.036), *rs3756963* (OR = 1.64, 95% CI = 1.14–2.37, *p*_*FDR*_ = 0.039) and *rs9468304* (OR = 1.75, 95% CI = 1.22–2.50, *p*_*FDR*_ = 0.017) were associated with an increased risk of ASD in the overdominant model. There were no associations for the remaining SNPs under any kind of genetic model.

**Table 3:** SNPs in significant genetic models associated with ASD risk

SNPs	Model	genotype	Control (243)	Case (243)	OR (95% CI)	*p*	*p*_FDR_
*rs174546*	overdominant	C/C-T/T	133 (55.6)	136 (56.7)	1.00	0.82	0.82
		T/C	106 (44.4)	104 (43.3)	0.96 (0.67–1.38)		
*rs174585*	dominant	G/G	199 (82.2)	210 (86.4)	1.00	0.2	0.375
		A/G-A/A	43 (17.8)	33 (13.6)	0.73 (0.44–1.19)		
*rs498793*	overdominant	C/C-T/T	210 (87.5%)	207 (85.9%)	1.00	0.6	0.75
		T/C	30 (12.5%)	34 (14.1%)	1.15 (0.68–1.95)		
*rs174602*	overdominant	T/T-C/C	145 (61.2)	157 (64.9)	1.00	0.4	0.667
		C/T	92 (38.8)	85 (35.1)	0.85 (0.59–1.24)		
*rs174593*	recessive	T/T-C/T	241 (99.2)	243 (100)	1.00	0.095	0.204
		C/C	2 (0.8)	0 (0)	NA		
*rs526126*	recessive	C/C-G/C	203 (83.5)	224 (98.7)	1.00	**< 0.0001**	< 0.01*
		G/G	40 (16.5)	3 (1.3)	**0.07 (0.02–0.22)**		
*rs174616*	overdominant	G/G-A/A	181 (74.5)	188 (77.7)	1.00	0.41	0.615
		A/G	62 (25.5)	54 (22.3)	0.84 (0.55–1.27)		
*rs17606561*	overdominant	G/G-A/A	164 (67.8)	137 (56.4)	1.00	**0.0096**	0.036*
		A/G	78 (32.2)	106 (43.6)	**1.63 (1.12–2.36)**		
*rs2236212*	recessive	C/C-G/C	212 (89.1)	210 (87.9)	1.00	0.68	0.729
		G/G	26 (10.9)	29 (12.1)	1.13 (0.64–1.98)		
*rs3798712*	overdominant	A/A-G/G	151 (63.5)	160 (66.1)	1.00	0.54	0.736
		G/A	87 (36.5)	82 (33.9)	0.89 (0.61–1.29)		
*rs953413*	recessive	A/A-G/A	241 (100)	232 (98.7)	1.00	0.039	0.098
		G/G	0 (0)	3 (1.3)	NA		
*rs3756963*	overdominant	T/T-C/C	161 (66.5)	133 (54.7)	1.00	**0.0078**	0.039*
		C/T	81 (33.5)	110 (45.3)	**1.64 (1.14–2.37)**		
*rs10498676*	recessive	G/G-A/G	184 (77)	204 (85.7)	1.00	**0.014**	0.042*
		A/A	55 (23)	34 (14.3)	**0.56 (0.35–0.89)**		
*rs6936315*	overdominant	T/T-C/C	141 (59.2)	149 (61.6)	1.00	0.6	0.692
		C/T	97 (40.8)	93 (38.4)	0.91 (0.63–1.31)		
*rs9468304*	overdominant	A/A-G/G	143 (59.1)	110 (45.3)	1.00	**0.0023**	0.017*
		G/A	99 (40.9)	133 (54.7)	**1.75 (1.22–2.50)**		

*OR* Odds ratio, *CI* confidence interval, *p*_FDR_ FDR corrected *p* value,* *p* < 0.05, *NA* is not applicable

### Haplotype analysis

**Fig. 2:**
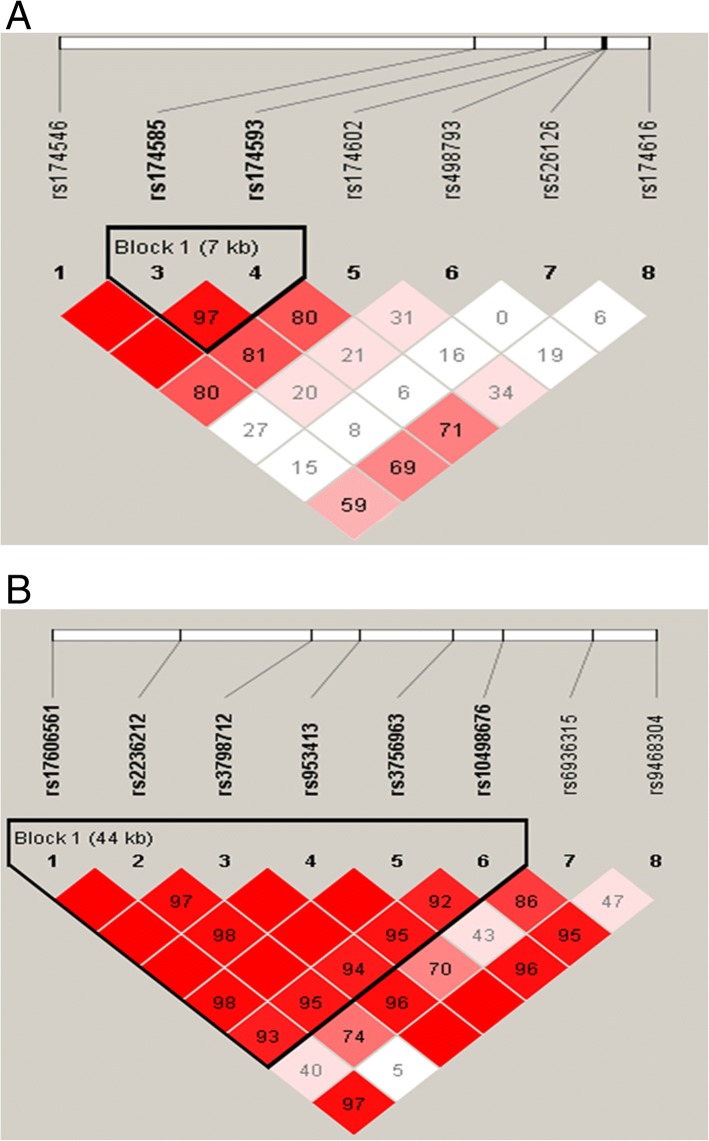
Haplotype block map for 7 SNPs of the *FADS1–2* gene cluster (**a**). Haplotype block map for 8 SNPs of the *ELOVL2* gene (**b**). The haplotypes were constructed based on the prevalence of individual SNPs and LD between them. Numbers in squares indicate D’ values. Explanation of color scheme: if D’ < 1 and LOD (log of the likelihood odds ratio) < 2, the cell is white; if D’ = 1 and LOD < 2, the cell color is blue; if D’ < 1 and LOD ≥ 2, the cell color is shades of pink or red; if D’ = 1 and LOD ≥ 2, the cell color is bright red

In the *FADS1/2* gene cluster, LD analysis of seven SNPs identified one haplotype block consisting of two SNPs (*rs174585* and *rs174593*) that produced two haplotypes with frequencies > 1% (Fig. 2a and Table 4). There were no differences in the frequencies of these haplotypes between ASD patients and controls.

**Table 4:** Distribution of *FADS1–2* and *ELOVL2* haplotypes in cases and controls (frequency more than 1%)

No.	Haplotype	Case ratio (243)	Control ratio (243)	*p*	OR (95 CI)
*FADS1–2*					
1	GT ^a^	0.926	0.903	0.207	1.00
2	AC ^a^	0.068	0.089	0.232	0.74(0.45–1.20)
*ELOVL2*					
1	GCAATA ^b^	0.380	0.439	0.057	1.00
2	ACAACG ^b^	0.253	0.203	0.066	1.46(1.04–2.05)
3	GGGATG ^b^	0.216	0.230	0.640	1.10(0.80–1.52)
4	GGAGTG ^b^	0.116	0.087	0.161	1.57(0.99–2.47)
5	GCAACG ^b^	0.010	0.017	0.404	0.72(0.23–2.25)

^a^ The order of SNPs in estimated analysis of haplotypes frequency: *rs174585* and *rs174593*

^b^ The order of SNPs in estimated analysis of haplotypes frequency: *rs17606561*, *rs2236212*, *rs3798712*, *rs953413*, *rs3756963* and *rs10498676*

In the *ELOVL2* gene, LD analysis of eight SNPs identified one haplotype block consisting of six SNPs (*rs17606561*, *rs2236212*, *rs3798712*, *rs953413*, *rs3756963*, and *rs10498676*) that produced five haplotypes with frequencies > 1 (Fig. 2b and Table 4). There were no differences in the frequencies of these haplotypes between ASD patients and controls.

### Association between genotype and ASD symptoms

According to above positive results, only 5 SNPs (*rs526126*, *rs17606561*, *rs3756963*, *rs10498676, rs9468304*) were selected in analyses of the relationships between gene polymorphisms and a series of ASD-specific features, including ADI-R and ADOS domains. Analysis of variance revealed that the A/A genotype of *rs10498676* had lower scores for ADI-R social, ADI-R verbal and nonverbal communication (Comm-V and -NV, respectively) than the G/G and A/G genotypes after adjusting for language level, IQ, sex and age. However, the positive result of ADI-R social presented no statistical significance after *Bonferroni* correction (Table 5). There were no associations for the remaining 4 SNPs (Additional file 1: Table S5).

**Table 5:** The genotype association of the *rs10498676* with ASD specific features in the recessive model

Items	n	G/G-A/G	A/A	*F*	*p*
ADI-R social	163	23.04±4.61	21.56±5.66	4.601	0.033
ADI-R comm-V	117	17.82±3.29	15.58±3.91	**9.221**	0.003*
ADI-R comm-NV	163	11.36±2.61	9.96±3.52	**8.814**	0.003*
ADI-R RR	163	5.43±2.77	6.04±2.89	0.335	0.564
ADOS-CSS	160	6.82±1.44	6.92±1.41	0.110	0.740
SA-CSS	160	7.16±1.57	7.27±1.49	0.113	0.737
RRB-CSS	160	6.39±1.88	6.38±1.77	0.000	0.993

*ADI-R* Autism Diagnostic Interview-Revised, *ADI-R comm-V* ADI-R communication for verbal, *ADI-R comm-NV* ADI-R communication for nonverbal, *ADI-R RR* ADI-R restricted repetitive behaviors, *ADOS* Autism Diagnostic Observation Schedule, *CSS* calibrated severity scores, *SA* social affect, *RRB* restricted repetitive behaviors, *F* statistic values from analysis of variance, * *p* < *p*_Bonferroni_ = 0.05/7

## Discussion

In this population-based case-control study, we aimed to investigate the effect of gene variations involved in LC-PUFA pathways and metabolism on the risk of ASD using allele, genotype, and haplotype analyses. We also investigated associations between gene polymorphisms and clinical features of ASD. This is the first study to investigate the association between *FADS1/2* and *ELOVL2* gene polymorphisms and ASD risk.

Our team previously found that significantly lower serum levels of AA, DHA and docosapentaenoic acid (DPA, 22:5n-3) in autistic children than in controls [14]. Then, the following animal experiments, we strictly controlled dietary conditions in both groups in order to reduce the effects of dietary factors, and we still found that serum n-3 and n-6 PUFA levels were lower in ASD model rats relative to controls. Moreover, we observed that the expression of rate-limiting PUFA metabolic enzymes was downregulated in the ASD group [18]. However, genetic mutations were not taken into consideration in our previous work. Investigating the role of genes involved in LC-PUFA metabolism may provide valuable insight into genetic susceptibility to ASD.

The *FADS1* and *FADS2* genes are seated head-to-head in a cluster on chromosome 11 (11q12–q13) and expressed in most human tissues, particularly in the liver, heart, and brain. An Avon longitudinal study in over 4000 pregnant women and their children indicated that *FADS* polymorphisms influenced maternal erythrocyte the concentrations of n-3 DHA, which disrupted the baby’s DHA supply in utero [23]. Animal studies have shown that irreversible and persistent changes in hypothalamic phospholipid composition resulted from abnormally high n-6:n-3 ratio early in life, the same as a dysfunction or down-regulation of the conversion of ALA to DHA by the delta-6 desaturase enzyme [24]. These two findings suggest that *FADS* polymorphisms may lead to irreversible structural and functional changes in neurons. In CHIANTI study reported that polymorphisms in the *FADS1/2* gene cluster accounted for as much as 18.6 of the variance in plasma and erythrocyte PUFA concentrations [25], and up to 28.5 of the variance in serum LC-PUFA content [9]. Moreover, the *FADS1/2* gene cluster is mainly involved in regulating the levels of pro- and anti-inflammatory eicosanoids synthesized from LC-PUFAs [26]. In our results, the frequencies of the minor allele G and genotype G/G of *rs526126* were lower in ASD cases than in controls, suggesting that individuals with the G allele have a lower risk of ASD. In other studies of this locus, *rs526126* G allele carriers had lower AA levels as well as reduced desaturase activity, as indicated by AA/dihomo-γ-linoleic acid (DGLA, 20:3n-6), γ-linoleic acid (GLA,18:3n-6)/LA, and AA/LA ratios [27]. The AA/DGLA, GLA/LA, and AA/LA product-to-precursor ratios are used to estimate FADS1, FADS2, and aggregate FADS1/2 desaturase indices, respectively. Moran et al. also found that the G allele of *rs526126* was nominally also related to lower levels of AA and lower FADS1 index, and the maternal G/G genotype was associated with higher child cognition scores, although this was not statistically significant [28]. In addition to the n-6 pathway, the minor allele G was positively associated with levels of desaturase subtrates (ALA) and negatively associated with levels of desaturase products (EPA and DHA) as well as ratios of n-3 (EPA/ALA) pathway components [23]. There are two possible explanations for the protective effects of allele G of *rs526126*. Firstly, eicosanoids, as the strongly pro-inflammatory factors, such as prostaglandins, thromboxanes, leukotrienes, and lipoxins that are transformed from AA via the cyclooxygenase and lipoxygenase pathways, are related to increased neuroinflammation, which is thought to damage nervous system development and has been associated with ASD [29]. Additionally, EPA can also produce eicosanoids, the lightly pro-inflammatory factors [26]. Secondly, endocannabinoids derived from PUFAs modulate synaptic function by suppressing neurotransmitter release, thereby acting as retrograde messengers in short-term forms of synaptic plasticity as well as presynaptically in long-term potentiation at both excitatory and inhibitory synapses [4, 11, 13, 30]. Genetic variant in *FADS2* (*rs526126)* may reduce desaturase activity, functionality, or expression, resulting in lower concentrations of LC-PUFAs and their derivatives, with downstream biological consequences.

*ELOVL2* is located on chromosome 6 (6p24.2) and encodes elongase 2, which has a key role in LC-PUFA biosynthesis. Although delta-6 desaturase has been accepted as a control point in the production of DHA from ALA, when dietary ALA or EPA is abundant, DHA levels do not increase, suggesting that there are other control points in the reactions of EPA to DHA. In particular, in the sequential reactions of EPA → DPA → tetracosapentaenoic acid (TPA, 24:5n-3), elongation of DPA to TPA by *ELOVL2* is a key step in the transformation of EPA to DHA [31]. Ablation of *Elovl2* in mice caused severe reduction in the levels of DHA and β-docosapentaenoic acid (BDPA, 22:5n-6), and accumulation of EPA, DPA and 22:4n-6 in both liver and serum [32]. SNPs in *ELOVL2* that reduce the efficiency of this process are expected to increase the production of DPA while decreasing that of TPA and DHA as the end product [31, 33]. Given the essential function of DHA in brain development and cognition-enhancing [4, 10], we speculated that SNPs in *ELOVL2* influence ASD susceptibility by modulating DHA synthesis. The current study showed that the allele distributions of tag SNPs in *ELOVL2* gene did not differ significantly between ASD cases and controls. However, the genotype analyses indicated four SNPs were significantly related to ASD risk in overdominate/recessive model. Given our somewhat conflicting observations, the relationship between *ELOVL2* gene and risk of ASD must be interpreted with cautious.

Except for rs17606561, most of SNPs in this study lied within intron, which performed vital roles in gene transcription. Mutations in introns, although non-coding sequences, can disturb splicing regulatory elements, such as enhancers or silencers, finally resulting in aberrant gene splicing [34]. It has been certified that the SNPs in the first intron of *FADS2* influenced *FADS1/2* expression [35]. The haplotyple block, as generally genetic unit, was conducted to evaluate the combined effect of the polymorphisms on ASD risk, and was an effective way of improving detection power comparing with single markers. Unfortunately, although haploview analyses demonstrated that strong LD between *rs174585* and *rs174593* in *FADS2* gene, and *rs17606561*, *rs2236212, rs3798712, rs953413, rs3756963* and *rs10498676* in *ELOVL2* gene (D’ = 0.97, mean D’ = 0.98, respectively), there were no marked association between the two reconstruction haplotype blocks of these tag SNPs in this study and ASD, which may have been a function of small size.

Estimating the severity of core autism features is critical for comparing samples across studies, tracking the course of the disorder over time, and quantifying treatment effects. However, the established measures for rating autism severity including the Childhood Autism Rating Scales, Autism Behavior Checklist, and raw ADOS scores yield variable results. This is may be attributable in part to differences in phenotypic characteristics such as IQ, language level, and chronological age, which affect the behavioral phenotypes of ASD and potentially masking the actual severity of core ASD symptoms. Gotham et al. addressed this heterogeneity by creating CSS based on raw total scores [19]. The CSS is relatively irrespective of the effect of phenotypic characteristics than the raw scores and allows scores to be compared across time, age, and modules. The use of ADOS and domain CSS has proven useful in characterizing samples for genetic and neurobiological studies [36, 37]. However, in the current literature we failed to uncover the relationship between *FADS2* and *ELOVL2* SNPs and ADOS and domain CSS. Our analysis revealed that *ELOVL2 rs10498676* is correlated with ADI-R comm-V and -NV after adjusting for age and language level. Compared with G/G and A/G genotypes, individuals with the A/A genotype had lower comm-V and -NV scores, which was in accordance with our previous finding that the minor A allele of *rs10498676* protected individuals against ASD. However, a caveat is that corrected ADI-R diagnostic scores may not represent the severity of ASD core symptoms.

The results of this study should be interpreted in light of several important limitations. Firstly, there were no data available regarding the LC-PUFA levels of the study subjects, and the relatively limited sample size decreased the power of the study. Secondly, both AIC and BIC can be derived from a model’s likelihood and resulting maximum likelihood estimate in the context of regression. Although AIC and BIC as the more general estimators can be used for model selection, this strategy could increase type I error due to their parsimony principle. To further verity or choose the optimally genetic model, diverse model selection strategies are worthy being performed. Finally, we did not include a replication cohort. Additional studies with larger samples and/or family-based association testing are needed to verify the precise contribution of gene polymorphisms to ASD.

## Conclusions

LC-PUFA metabolism is an important area of ASD research, given their roles in brain development and in the modulation of inflammatory response in the brain. Our results demonstrate that *FADS2* and *ELOVL2* SNPs differed between ASD cases and controls and suggest that these genes contribute to genetic susceptibility to ASD in a Chinese Han population. These findings provide new evidence for the contribution of genetic susceptibility to the etiology of ASD.

## Additional file

Additional file 1: **Table S1.** Sample description and scores on primary measures. **Table S2**. General information of gene tag SNPs. **Table S3**. Primers used in the screening of SNPs by MassArray. **Table S4**. SNPs in different genetic models associated with ASD risk. **Table S5.** The genotype association of the 4 SNPs with ASD specific features in the significant genetic model. (DOCX 73 kb)

## Abbreviations

AA: Arachidonic acid
ADI-R comm-NV: ADI-R communication for nonverbal
ADI-R comm-V: ADI-R communication for verbal
ADI-R RR: ADI-R restricted repetitive behaviors
ADI-R: Autism Diagnostic Interview-Revised
ADOS: Autism Diagnostic Observation Schedule
AIC: Akaike’ information criterion
ALA: Alpha-linolenic acid
ASD: Autism Spectrum Disorders
BDPA: β-docosapentaenoic acid
BIC: Bayesian information criterion
CHB: Chinese Han in Beijing
CI: Confidence interval
CSS: Calibrated severity scores
DGLA: Dihomo-γ-linolenicacid
DHA: Docosahexanoic acid
DPA: Docosapentaenoic acid
DSM-V: Diagnostic and Statistical Manual of Mental Disorders, Fifth Edition
ELOVL2: Elongation of very long chain fatty acids 2/elongase 2
EPA: Eicosapentaenoic acid
FADS1: Fatty acid desaturase 1/delta-5-desaturase
FADS2: Fatty acid desaturase 2/ delta-6-desaturase
GLA: γ-linolenicacid
HWE: Hardy-Weinberg equilibrium
IQ: Intelligence quotient
LA: Linoleic acid
LD: Linkage disequilibrium
MAF: Minor Allele Frequency
OR: Odd ratio
PUFA: Polyunsaturated fatty acid
RRB: Restricted repetitive behaviors
SA: Social affect
SNP: Single nucleotide polymorphism
TPA: Tetracosapentaenoic acid
