# Supplementary material for: FADS1-FADS2 and ELOVL2 gene polymorphisms in susceptibility to autism spectrum disorders in Chinese children
Source: BMC Psychiatry. 2018 Sep 4;18:283. doi: 10.1186/s12888-018-1868-7 (PMC6122697; doi:10.1186/s12888-018-1868-7)
Supplement: Supplementary file 1 — Table S1. Sample description and scores on primary measures. Table S2. General information of gene tag SNPs. Table S3. Primers used in the screening of SNPs by MassArray. Table S4. SNPs in different genetic models associated with ASD risk. Table S5. The genotype association of the 4 SNPs with ASD specific features in the significant genetic model. (DOCX 73 kb) [file 12888_2018_1868_MOESM1_ESM.docx]

**Table S1. Sample description and scores on primary measures**

| Variants | n | mean±SD, n (%) |
| --- | --- | --- |
| Chronological age (years) | 184 | 5.45±1.96 |
| Gender (male) | 184 | 153 (83.2) |
| IQ | 183 | 53.78±21.64 |
| ADOS | 174 |  |
| SA-CSS | 165 | 7.10±1.62 |
| RRB-CSS | 165 | 6.38±1.89 |
| ADOS-CSS | 165 | 6.76±1.52 |
| ADI-R | 176 |  |
| ADI- social | 167 | 22.81±4.75 |
| ADI-R comm-V | 120 | 17.48±3.47 |
| ADI-R comm-NV | 167 | 11.11±2.79 |
| ADI-R RR | 167 | 5.48±2.79 |

IQ = intelligence quotients, ADOS=Autism Diagnostic Observation Schedule, SA = social affect, RRB = restricted repetitive behavior, CSS = calibrated severity scores, ADI-R = Autism Diagnostic Interview-Revised, ADI-R comm-V, ADI-R communication for verbal; ADI-R comm-NV, ADI-R communication for nonverbal; ADI-R RR, ADI-R restricted repetitive behaviors;

**Table S2. General information of gene tag SNPs**

| **SNP ID** | **Gene** | **Genomic position (bp)** | **Genic position** | **Minor Allele^a^** | **MAF^a^** | **HWE(p)^a^** |
| --- | --- | --- | --- | --- | --- | --- |
| *rs174546* | *FADS1* | 61802358 | 3’UTR | T | 0.341 | 0.728 |
| *rs2845573* | *FADS2* | 61834436 | intron | G | 0.233 | 0.333 |
| *rs174585* | *FADS2* | 61844222 | intron | A | 0.105 | 0.672 |
| *rs174593* | *FADS2* | 61851359 | intron | C | 0.091 | 0.588 |
| *rs174602* | *FADS2* | 61856942 | intron | C | 0.273 | 0.782 |
| *rs498793* | *FADS2* | 61857233 | intron | T | 0.100 | 0.715 |
| *rs526126* | *FADS2* | 61857413 | intron | G | 0.102 | 1.000 |
| *rs174616* | *FADS2* | 61861650 | intron | A | 0.205 | 1.000 |
| *rs17606561* | *ELOVL2* | 10982126 | 3’UTR | A | 0.171 | 1.000 |
| *rs2236212* | *ELOVL2* | 10994782 | intron | C | 0.300 | 1.000 |
| *rs3798712* | *ELOVL2* | 11007869 | intron | G | 0.222 | 1.000 |
| *rs953413* | *ELOVL2* | 11012626 | intron | G | 0.078 | 1.000 |
| *rs3756963* | *ELOVL2* | 11021921 | intron | C | 0.278 | 0.525 |
| *rs10498676* | *ELOVL2* | 11026766 | intron | A | 0.478 | 1.000 |
| *rs6936315* | *ELOVL2* | 11035739 | intron | C | 0.278 | 0.525 |
| *rs9468304* | *ELOVL2* | 11041932 | intron | A | 0.311 | 0.611 |

MAF, minor allele frequency; HWE, Hardy-Weinberg equilibrium; ^a^ among CHB in HAPMAP database.

**Table S3.** **Primers used in the screening of SNPs by MassArray**

| **SNPs** |  | **Primers** | **Amplicon size(bp)** |
| --- | --- | --- | --- |
| *rs174546* | Forward | ACGTTGGATGTAGTGGCATTGTCCCTCAAG | 109 |
|  | Reverse | ACGTTGGATGAATAACAACAGCCACCCTGC |  |
| *rs2845573* | Forward | ACGTTGGATGTCTTCACCTCAGAAGTTGGG | 106 |
|  | Reverse | ACGTTGGATGGGTAATGGGCATGCGCCAG |  |
| *rs174593* | Forward | ACGTTGGATGAACAACCAGATCCTCACAGC | 109 |
|  | Reverse | ACGTTGGATGTTTCCCGACATCTTCTCACC |  |
| *rs498793* | Forward | ACGTTGGATGCTAAACTTGTTAGAAGCGGG | 115 |
|  | Reverse | ACGTTGGATGACTCTCTGAGCCTCTGTGTC |  |
| *rs174602* | Forward | ACGTTGGATGAGATCATGAGCACCCAGTAG | 103 |
|  | Reverse | ACGTTGGATGAGCAGATAGAAGGGATGGTG |  |
| *rs526126* | Forward | ACGTTGGATGAACTGCAGGAGAGAGACAGG | 94 |
|  | Reverse | ACGTTGGATGCTGGGTTCCCCTGACCTTC |  |
| *rs174616* | Forward | ACGTTGGATGTCAGGAGAGGCTGGAGATG | 102 |
|  | Reverse | ACGTTGGATGCACCTTGAAGGCCACCTTAT |  |
| *rs174585* | Forward | ACGTTGGATGACATTCTAAGTGTAAATGC | 114 |
|  | Reverse | ACGTTGGATGGCCCGGTCTTAACTAATTTC |  |
| *rs17606561* | Forward | ACGTTGGATGCACACAGGAGACATCTGTTC | 81 |
|  | Reverse | ACGTTGGATGACCTGCTTCCTTCCCTTCAG |  |
| *rs2236212* | Forward | ACGTTGGATGGATACAGGCAAAATCTGGGC | 95 |
|  | Reverse | ACGTTGGATGTGACCATGGGACATATGATG |  |
| *rs3798712* | Forward | ACGTTGGATGTTACAGCACTGTGACAAAAG | 98 |
|  | Reverse | ACGTTGGATGTGGCACATTGAGTCAAGCAC |  |
| *rs953413* | Forward | ACGTTGGATGTTTCTGCCCTTCTTCCACTG | 101 |
|  | Reverse | ACGTTGGATGGCTAAGAAAACGCTAAAGGTC |  |
| *rs3756963* | Forward | ACGTTGGATGCTTTGTGCGAGAACCTGAGC | 100 |
|  | Reverse | ACGTTGGATGCTGTCAGCATCTGACACTCC |  |
| *rs10498676* | Forward | ACGTTGGATGGTATGCAGCATATGGAATAG | 113 |
|  | Reverse | ACGTTGGATGCTCCATATAGCTATGTAAGG |  |
| *rs6936315* | Forward | ACGTTGGATGCACAAGTCAGCAGTGTTTGG | 91 |
|  | Reverse | ACGTTGGATGTTGGATGGAATGGCACATGG |  |
| *rs9468304* | Forward | ACGTTGGATGGCAGAGATATCTTACAAGAC | 94 |
|  | Reverse | ACGTTGGATGCTTAGTGGGTCAGTTCAGAG |  |

**Table S4. SNPs in different genetic models associated with ASD risk**

| **SNPs** | **Model** | **genotype** | **Control (243)** | **Case (243)** | **OR (95% CI)** | ***p*** | **AIC** | | **BIC** | ***p* _FDR_** |
| --- | --- | --- | --- | --- | --- | --- | --- | --- | --- | --- |
| *rs174546* | codominant | C/C | 109(45.6) | 112(46.7) | 1.00 | 0.97 | 670 | | 682.5 |  |
|  |  | T/C | 106(44.4) | 104(43.3) | 0.95 (0.65-1.39) |  |  |  |  |  |
|  |  | T/T | 24(10.0) | 24(10.0) | 0.97 (0.52-1.82) |  |  |  |  |  |
|  | dominant | C/C | 109(45.6) | 112(46.7) | 1.00 | 0.82 | 668 | | 676.3 |  |
|  |  | T/C-T/T | 130(54.4) | 128(53.3) | 0.96 (0.67-1.37) |  |  |  |  |  |
|  | recessive | C/C-T/C | 215 (90) | 216 (90) | 1.00 | 0.99 | 668 | | 676.4 |  |
|  |  | T/T | 24 (10) | 24 (10) | 1.00 (0.55-1.81) |  |  |  |  |  |
|  | overdominant | C/C-T/T | 133 (55.6) | 136 (56.7) | 1.00 | 0.82 | 668 | | 676.3 | 0.82 |
|  |  | T/C | 106 (44.4) | 104 (43.3) | 0.96 (0.67-1.38) |  |  |  |  |  |
|  | Log-additive | --- | --- | --- | 0.97 (0.74-1.28) | 0.85 | 668 | | 676.3 |  |
| *rs174585* | codominant | G/G | 199(82.2) | 210(86.4) | 1.00 | 0.44 | 676.7 | | 689.3 |  |
|  |  | A/G | 42(17.4) | 32(13.2) | 0.72 (0.44-1.19) |  |  |  |  |  |
|  |  | A/A | 1(0.4) | 1(0.4) | 0.95 (0.06-15.25) |  |  |  |  |  |
|  | dominant | G/G | 199 (82.2) | 210 (86.4) | 1.00 | 0.2 | 674.7 | | 683.1 | 0.375 |
|  |  | A/G-A/A | 43 (17.8) | 33 (13.6) | 0.73 (0.44-1.19) |  |  |  |  |  |
|  | recessive | G/G-A/G | 241 (99.6) | 242 (99.6) | 1.00 | 1 | 676.4 | | 684.7 |  |
|  |  | A/A | 1 (0.4) | 1 (0.4) | 1.00 (0.06-16.01) |  |  |  |  |  |
|  | overdominant | G/G-A/A | 200 (82.6) | 211 (86.8) | 1.00 | 0.2 | 674.7 | | 683.1 |  |
|  |  | A/G | 42 (17.4) | 32 (13.2) | 0.72 (0.44-1.19) |  |  |  |  |  |
|  | Log-additive | --- | --- | --- | 0.75 (0.46-1.20) | 0.22 | 674.9 | | 683.2 |  |
| *rs498793* | codominant | C/C | 207 (86.2%) | 205 (85.1%) | 1.00 | 0.79 | 672.3 | | 684.9 |  |
|  |  | T/C | 30 (12.5%) | 34 (14.1%) | 1.14 (0.68-1.94) |  |  |  |  |  |
|  |  | T/T | 3 (1.2%) | 2 (0.8%) | 0.67 (0.11-4.07) |  |  |  |  |  |
|  | dominant | C/C | 207 (86.2%) | 205 (85.1%) | 1.00 | 0.71 | 670.7 | | 679 |  |
|  |  | T/C-T/T | 33 (13.8%) | 36 (14.9%) | 1.10 (0.66-1.83) |  |  |  |  |  |
|  | recessive | C/C-T/C | 237 (98.8%) | 239 (99.2%) | 1.00 | 0.65 | 670.6 | | 678.9 |  |
|  |  | T/T | 3 (1.2%) | 2 (0.8%) | 0.66 (0.11-3.99) |  |  |  |  |  |
|  | overdominant | C/C-T/T | 210 (87.5%) | 207 (85.9%) | 1.00 | 0.6 | 670.5 | | 678.9 | 0.75 |
|  |  | T/C | 30 (12.5%) | 34 (14.1%) | 1.15 (0.68-1.95) |  |  |  |  |  |
|  | Log-additive | --- | --- | --- | 1.05 (0.66-1.67) | 0.83 | 670.8 | | 679.1 |  |
| *rs174602* | codominant | T/T | 134 (56.5) | 145 (59.9) | 1.00 | 0.7 | 669.3 | | 681.8 |  |
|  |  | C/T | 92 (38.8) | 85 (35.1) | 0.85 (0.59-1.24) |  |  |  |  |  |
|  |  | C/C | 11 (4.6%) | 12 (5%) | 1.01 (0.43-2.36) |  |  |  |  |  |
|  | dominant | T/T | 134 (56.5%) | 145 (59.9%) | 1.00 | 0.45 | 667.4 | | 675.8 |  |
|  |  | C/T-C/C | 103 (43.5%) | 97 (40.1%) | 0.87 (0.61-1.25) |  |  |  |  |  |
|  | recessive | T/T-C/T | 226 (95.4%) | 230 (95%) | 1.00 | 0.87 | 668 | | 676.3 |  |
|  |  | C/C | 11 (4.6) | 12 (5) | 1.07 (0.46-2.48) |  |  |  |  |  |
|  | overdominant | T/T-C/C | 145 (61.2) | 157 (64.9) | 1.00 | 0.4 | 667.3 | | 675.6 | 0.667 |
|  |  | C/T | 92 (38.8) | 85 (35.1) | 0.85 (0.59-1.24) |  |  |  |  |  |
|  | Log-additive | --- | --- | --- | 0.92 (0.67-1.24) | 0.57 | 667.7 | | 676 |  |
| *rs174593* | codominant | T/T | 199 (81.9) | 208 (85.6) | 1.00 | 0.16 | 676.1 | | 688.7 |  |
|  |  | C/T | 42 (17.3) | 35 (14.4) | 0.80 (0.49-1.30) |  |  |  |  |  |
|  |  | C/C | 2 (0.8) | 0 (0) | NA |  |  |  |  |  |
|  | dominant | T/T | 199 (81.9) | 208 (85.6) | 1.00 | 0.27 | 676.5 | | 684.9 |  |
|  |  | C/T-C/C | 44 (18.1) | 35 (14.4) | 0.76 (0.47-1.24) |  |  |  |  |  |
|  | recessive | T/T-C/T | 241 (99.2) | 243 (100) | 1.00 | 0.095 | 675 | | 683.3 | 0.204 |
|  |  | C/C | 2 (0.8) | 0 (0) | NA |  |  |  |  |  |
|  | overdominant | T/T-C/C | 201 (82.7) | 208 (85.6) | 1.00 | 0.38 | 677 | | 685.4 |  |
|  |  | C/T | 42 (17.3) | 35 (14.4) | 0.81 (0.49-1.31) |  |  |  |  |  |
|  | Log-additive | --- | --- | --- | 0.73 (0.46-1.17) | 0.19 | 676 | | 684.4 |  |
| *rs526126* | codominant | C/C | 72 (29.6) | 91 (40.1) | 1.00 | <0.0001 | 617.5 | | 629.9 |  |
|  |  | G/C | 131 (53.9) | 133 (58.6) | 0.80 (0.54-1.19) |  |  |  |  |  |
|  |  | G/G | 40 (16.5) | 3 (1.3) | 0.06 (0.02-0.20) |  |  |  |  |  |
|  | dominant | C/C | 72 (29.6) | 91 (40.1) | 1.00 | 0.017 | 649.3 | | 657.6 |  |
|  |  | G/C-G/G | 171 (70.4) | 136 (59.9) | 0.63 (0.43-0.92) |  |  |  |  |  |
|  | recessive | C/C-G/C | 203 (83.5) | 224 (98.7) | 1.00 | **<0.0001** | 616.7 | | 625 | **<0.01*** |
|  |  | G/G | 40 (16.5) | 3 (1.3) | **0.07 (0.02-0.22)** |  |  |  |  |  |
|  | overdominant | C/C-G/G | 112 (46.1) | 94 (41.4) | 1.00 | 0.31 | 654 | | 662.3 |  |
|  |  | G/C | 131 (53.9) | 133 (58.6) | 1.21 (0.84-1.74) |  |  |  |  |  |
|  | Log-additive | --- | --- | --- | 0.49 (0.36-0.67) | <0.0001 | 634 | | 642.3 |  |
| *rs174616* | codominant | G/G | 175 (72) | 180 (74.4) | 1.00 | 0.64 | 677.4 | | 690 |  |
|  |  | A/G | 62 (25.5) | 54 (22.3) | 0.85 (0.56-1.29) |  |  |  |  |  |
|  |  | A/A | 6 (2.5) | 8 (3.3) | 1.30 (0.44-3.81) |  |  |  |  |  |
|  | dominant | G/G | 175 (72) | 180 (74.4) | 1.00 | 0.56 | 676 | | 684.4 |  |
|  |  | A/G-A/A | 68 (28) | 62 (25.6) | 0.89 (0.59-1.33) |  |  |  |  |  |
|  | recessive | G/G-A/G | 237 (97.5) | 234 (96.7) | 1.00 | 0.58 | 676 | | 684.4 |  |
|  |  | A/A | 6 (2.5) | 8 (3.3) | 1.35 (0.46-3.95) |  |  |  |  |  |
|  | overdominant | G/G-A/A | 181 (74.5) | 188 (77.7) | 1.00 | 0.41 | 675.7 | | 684 | 0.615 |
|  |  | A/G | 62 (25.5) | 54 (22.3) | 0.84 (0.55-1.27) |  |  |  |  |  |
|  | Log-additive | --- | --- | --- | 0.94 (0.67-1.33) | 0.74 | 676.2 | | 684.6 |  |
| *rs17606561* | codominant | G/G | 151 (62.4) | 127 (52.3) | 1.00 | 0.034 | 671.6 | | 684.2 |  |
|  |  | A/G | 78 (32.2) | 106 (43.6) | 1.62 (1.11-2.35) |  |  |  |  |  |
|  |  | A/A | 13 (5.4) | 10 (4.1) | 0.91 (0.39-2.16) |  |  |  |  |  |
|  | dominant | G/G | 151 (62.4) | 127 (52.3) | 1.00 | 0.024 | 671.3 | | 679.6 |  |
|  |  | A/G-A/A | 91 (37.6) | 116 (47.7) | 1.52 (1.06-2.18) |  |  |  |  |  |
|  | recessive | G/G-A/G | 229 (94.6) | 233 (95.9) | 1.00 | 0.51 | 675.9 | | 684.3 |  |
|  |  | A/A | 13 (5.4) | 10 (4.1) | 0.76 (0.32-1.76) |  |  |  |  |  |
|  | overdominant | G/G-A/A | 164 (67.8) | 137 (56.4) | 1.00 | **0.0096** | 669.7 | | 678 | **0.036*** |
|  |  | A/G | 78 (32.2) | 106 (43.6) | **1.63 (1.12-2.36)** |  |  |  |  |  |
|  | Log-additive | --- | --- | --- | 1.30 (0.95-1.76) | 0.095 | 673.6 | | 681.9 |  |
| *rs2236212* | codominant | C/C | 106 (44.5) | 107 (44.8) | 1.00 | 0.9 | 667.1 | | 679.6 |  |
|  |  | G/C | 106 (44.5) | 103 (43.1) | 0.96 (0.66-1.41) |  |  |  |  |  |
|  |  | G/G | 26 (10.9) | 29 (12.1) | 1.10 (0.61-2.00) |  |  |  |  |  |
|  | dominant | C/C | 106 (44.5) | 107 (44.8) | 1.00 | 0.96 | 665.3 | | 673.6 |  |
|  |  | G/C-G/G | 132 (55.5) | 132 (55.2) | 0.99 (0.69-1.42) |  |  |  |  |  |
|  | recessive | C/C-G/C | 212 (89.1) | 210 (87.9) | 1.00 | 0.68 | 665.1 | | 673.4 | 0.729 |
|  |  | G/G | 26 (10.9) | 29 (12.1) | 1.13 (0.64-1.98) |  |  |  |  |  |
|  | overdominant | C/C-G/G | 132 (55.5) | 136 (56.9) | 1.00 | 0.75 | 665.2 | | 673.5 |  |
|  |  | G/C | 106 (44.5) | 103 (43.1) | 0.94 (0.66-1.35) |  |  |  |  |  |
|  | Log-additive | --- | --- | --- | 1.02 (0.78-1.33) | 0.87 | 665.2 | | 673.6 |  |
| *rs3798712* | codominant | A/A | 139 (58.4) | 146 (60.3) | 1.00 | 0.8 | 670.9 | | 683.5 |  |
|  |  | G/A | 87 (36.5) | 82 (33.9) | 0.90 (0.61-1.31) |  |  |  |  |  |
|  |  | G/G | 12 (5) | 14 (5.8) | 1.11 (0.50-2.49) |  |  |  |  |  |
|  | dominant | A/A | 139 (58.4) | 146 (60.3) | 1.00 | 0.67 | 669.2 | | 677.6 |  |
|  |  | G/A-G/G | 99 (41.6) | 96 (39.7) | 0.92 (0.64-1.33) |  |  |  |  |  |
|  | recessive | A/A-G/A | 226 (95) | 228 (94.2) | 1.00 | 0.72 | 669.3 | | 677.6 |  |
|  |  | G/G | 12 (5) | 14 (5.8) | 1.16 (0.52-2.55) |  |  |  |  |  |
|  | overdominant | A/A-G/G | 151 (63.5) | 160 (66.1) | 1.00 | 0.54 | 669 | | 677.4 | 0.736 |
|  |  | G/A | 87 (36.5) | 82 (33.9) | 0.89 (0.61-1.29) |  |  |  |  |  |
|  | Log-additive | --- | --- | --- | 0.97 (0.72-1.31) | 0.83 | 669.3 | | 677.7 |  |
| *rs953413* | codominant | A/A | 195 (80.9) | 183 (77.9) | 1.00 | 0.1 | 661.2 | | 673.7 |  |
|  |  | G/A | 46 (19.1) | 49 (20.9) | 1.14 (0.72-1.78) |  |  |  |  |  |
|  |  | G/G | 0 (0) | 3 (1.3) | NA |  |  |  |  |  |
|  | dominant | A/A | 195 (80.9) | 183 (77.9) | 1.00 | 0.41 | 663.1 | | 671.5 |  |
|  |  | G/A-G/G | 46 (19.1) | 52 (22.1) | 1.20 (0.77-1.88) |  |  |  |  |  |
|  | recessive | A/A-G/A | 241 (100) | 232 (98.7) | 1.00 | 0.039 | 659.5 | | 667.9 | 0.098 |
|  |  | G/G | 0 (0) | 3 (1.3) | NA |  |  |  |  |  |
|  | overdominant | A/A-G/G | 195 (80.9) | 186 (79.2) | 1.00 | 0.63 | 663.6 | | 671.9 |  |
|  |  | G/A | 46 (19.1) | 49 (20.9) | 1.12 (0.71-1.75) |  |  |  |  |  |
|  | Log-additive | --- | --- | --- | 1.27 (0.83-1.95) | 0.27 | 662.6 | | 670.9 |  |
| *rs3756963* | codominant | T/T | 146 (60.3) | 122 (50.2) | 1.00 | 0.028 | 671.2 | | 683.7 |  |
|  |  | C/T | 81 (33.5) | 110 (45.3) | 1.63 (1.12-2.36) |  |  |  |  |  |
|  |  | C/C | 15 (6.2) | 11 (4.5) | 0.88 (0.39-1.98) |  |  |  |  |  |
|  | dominant | T/T | 146 (60.3) | 122 (50.2) | 1.00 | 0.025 | 671.3 | | 679.7 |  |
|  |  | C/T-C/C | 96 (39.7) | 121 (49.8) | 1.51 (1.05-2.16) |  |  |  |  |  |
|  | recessive | T/T-C/T | 227 (93.8) | 232 (95.5) | 1.00 | 0.41 | 675.7 | | 684 |  |
|  |  | C/C | 15 (6.2) | 11 (4.5) | 0.72 (0.32-1.60) |  |  |  |  |  |
|  | overdominant | T/T-C/C | 161 (66.5) | 133 (54.7) | 1.00 | **0.0078** | 669.3 | | 677.6 | **0.039*** |
|  |  | C/T | 81 (33.5) | 110 (45.3) | **1.64 (1.14-2.37)** |  |  |  |  |  |
|  | Log-additive | --- | --- | --- | 1.27 (0.94-1.71) | 0.12 | 673.9 | | 682.3 |  |
| *rs10498676* | codominant | G/G | 79 (33) | 85 (35.7) | 1.00 | 0.048 | 661.2 | | 673.7 |  |
|  |  | A/G | 105 (43.9) | 119 (50) | 1.05 (0.70-1.58) |  |  |  |  |  |
|  |  | A/A | 55 (23) | 34 (14.3) | 0.57 (0.34-0.97) |  |  |  |  |  |
|  | dominant | G/G | 79 (33) | 85 (35.7) | 1.00 | 0.54 | 664.9 | | 673.2 |  |
|  |  | A/G-A/A | 160 (67) | 153 (64.3) | 0.89 (0.61-1.30) |  |  |  |  |  |
|  | recessive | G/G-A/G | 184 (77) | 204 (85.7) | 1.00 | **0.014** | 659.2 | | 667.6 | **0.042*** |
|  |  | A/A | 55 (23) | 34 (14.3) | **0.56 (0.35-0.89)** |  |  |  |  |  |
|  | overdominant | G/G-A/A | 134 (56.1) | 119 (50) | 1.00 | 0.18 | 663.5 | | 671.8 |  |
|  |  | A/G | 105 (43.9) | 119 (50) | 1.28 (0.89-1.83) |  |  |  |  |  |
|  | Log-additive | --- | --- | --- | 0.80 (0.62-1.03) | 0.08 | 662.2 | | 670.5 |  |
| *rs6936315* | codominant | T/T | 121 (50.8) | 128 (52.9) | 1.00 | 0.87 | 671.1 | | 683.6 |  |
|  |  | C/T | 97 (40.8) | 93 (38.4) | 0.91 (0.62-1.32) |  |  |  |  |  |
|  |  | C/C | 20 (8.4) | 21 (8.7) | 0.99 (0.51-1.92) |  |  |  |  |  |
|  | dominant | T/T | 121 (50.8) | 128 (52.9) | 1.00 | 0.65 | 669.2 | 677.5 | |  |
|  |  | C/T-C/C | 117 (49.2) | 114 (47.1) | 0.92 (0.64-1.32) |  |  |  |  |  |
|  | recessive | T/T-C/T | 218 (91.6) | 221 (91.3) | 1.00 | 0.91 | 669.4 | | 677.7 |  |
|  |  | C/C | 20 (8.4) | 21 (8.7) | 1.04 (0.55-1.96) |  |  |  |  |  |
|  | overdominant | T/T-C/C | 141 (59.2) | 149 (61.6) | 1.00 | 0.6 | 669.1 | | 677.5 | 0.692 |
|  |  | C/T | 97 (40.8) | 93 (38.4) | 0.91 (0.63-1.31) |  |  |  |  |  |
|  | Log-additive | --- | --- | --- | 0.96 (0.73-1.26) | 0.76 | 669.3 | | 677.6 |  |
| *rs9468304* | codominant | A/A | 115 (47.5) | 83 (34.2) | 1.00 | 0.0061 | 668.1 | | 680.7 |  |
|  |  | G/A | 99 (40.9) | 133 (54.7) | 1.86 (1.27-2.73) |  |  |  |  |  |
|  |  | G/G | 28 (11.6) | 27 (11.1) | 1.34 (0.73-2.43) |  |  |  |  |  |
|  | dominant | A/A | 115 (47.5) | 83 (34.2) | 1.00 | 0.0027 | 667.4 | | 675.7 |  |
|  |  | G/A-G/G | 127 (52.5) | 160 (65.8) | 1.75 (1.21-2.52) |  |  |  |  |  |
|  | recessive | A/A-G/A | 214 (88.4) | 216 (88.9) | 1.00 | 0.87 | 676.3 | | 684.7 |  |
|  |  | G/G | 28 (11.6) | 27 (11.1) | 0.96 (0.54-1.67) |  |  |  |  |  |
|  | overdominant | A/A-G/G | 143 (59.1) | 110 (45.3) | 1.00 | **0.0023** | 667 | | 675.4 | **0.017*** |
|  |  | G/A | 99 (40.9) | 133 (54.7) | **1.75 (1.22-2.50)** |  |  |  |  |  |
|  | Log-additive | --- | --- | --- | 1.35 (1.03-1.77) | 0.031 | 671.7 | | 680.1 |  |

OR, Odds ratio; CI, confidence interval; AIC, Akaike’ information criterion; BIC, Bayesian information criterion; *p*_FDR_: FDR corrected p value; * *p* < 0.05; NA is not applicable.

**Table S5. The genotype association of the 4 SNPs with ASD specific features in the significant genetic model.**

| **SNP** | **items** | **n** | **genotype** | **genotype** | ***F*** | ***p*** |
| --- | --- | --- | --- | --- | --- | --- |
| *rs526126* |  |  | C/C-C/G | G/G |  |  |
|  | ADI- social | 156 | 22.84±4.86 | 22.67±3.51 | 0.018 | 0.894 |
|  | ADI-R comm-V | 111 | 17.37±3.49 | 18.00±4.24 | 0.089 | 0.766 |
|  | ADI-R comm-NV | 156 | 11.11±2.80 | 9.67±3.06 | 0.867 | 0.353 |
|  | ADI-R RR | 156 | 5.48±2.84 | 5.67±1.53 | 0.085 | 0.771 |
|  | ADOS-CSS | 152 | 6.83±1.45 | 7.67±0.58 | 0.980 | 0.324 |
|  | SA-CSS | 152 | 7.16±1.56 | 7.33±1.53 | 0.036 | 0.850 |
|  | RRB-CSS | 152 | 6.34±1.90 | 7.67±1.16 | 1.443 | 0.232 |
| *rs17606561* |  |  | G/G-A/A | A/G |  |  |
|  | ADI-R social | 167 | 22.55±4.72 | 23.17±4.80 | 0.491 | 0.484 |
|  | ADI-R comm-V | 120 | 17.37±3.61 | 17.67±3.24 | 0.890 | 0.347 |
|  | ADI-R comm-NV | 167 | 10.96±2.86 | 11.33±2.69 | 0.371 | 0.543 |
|  | ADI-R RR | 167 | 5.44±2.84 | 5.54±2.74 | 0.008 | 0.928 |
|  | ADOS-CSS | 165 | 6.78±1.39 | 6.90±1.46 | 0.314 | 0.576 |
|  | SA-CSS | 165 | 7.11±1.51 | 7.27±1.58 | 0.443 | 0.507 |
|  | RRB-CSS | 165 | 6.22±1.92 | 6.61±1.73 | 1.747 | 0.188 |
| *rs3756963* |  |  | T/T-C/C | C/T |  |  |
|  | ADI-R social | 167 | 22.52±4.74 | 23.19±4.76 | 0.771 | 0.381 |
|  | ADI-R comm-V | 120 | 17.34±3.65 | 17.70±3.19 | 1.211 | 0.273 |
|  | ADI-R comm-NV | 167 | 11.01±2.86 | 11.25±2.71 | 0.206 | 0.650 |
|  | ADI-R RR | 167 | 5.41±2.80 | 5.57±2.79 | 0.025 | 0.874 |
|  | ADOS-CSS | 165 | 6.87±1.24 | 6.78±1.61 | 0.160 | 0.690 |
|  | SA-CSS | 165 | 7.20±1.40 | 7.15±1.71 | 0.035 | 0.853 |
|  | RRB-CSS | 165 | 6.27±1.91 | 6.53±1.76 | 0.825 | 0.365 |
| *rs9468304* |  |  | A/A-G/G | G/A |  |  |
|  | ADI-R social | 167 | 22.33±4.77 | 23.29±4.71 | 1.643 | 0.202 |
|  | ADI-R comm-V | 120 | 17.05±3.70 | 17.95±3.17 | **4.251** | **0.041** |
|  | ADI-R comm-NV | 167 | 10.82±2.92 | 11.40±2.64 | 1.474 | 0.226 |
|  | ADI-R RR | 167 | 5.51±2.81 | 5.45±2.78 | 0.215 | 0.644 |
|  | ADOS-CSS | 165 | 6.77±1.36 | 6.89±1.47 | 0.275 | 0.601 |
|  | SA-CSS | 165 | 7.10±1.50 | 7.24±1.58 | 0.334 | 0.564 |
|  | RRB-CSS | 165 | 6.15±2.04 | 6.60±1.63 | 2.405 | 0.123 |

ADI-R, Autism Diagnostic Interview-Revised; ADI-R comm-V, ADI-R communication for verbal; ADI-R comm-NV, ADI-R communication for nonverbal; ADI-R RR, ADI-R restricted repetitive behaviors; ADOS, Autism Diagnostic Observation Schedule; CSS, calibrated severity scores; SA, social affect; RRB, restricted repetitive behaviors; *F*, statistic values from analysis of variance.
